# Clinical and radiographic success of bioflex versus stainless steel crowns using the hall technique in primary molars: a 12-month randomized controlled trial

**DOI:** 10.1007/s00784-026-07156-4

**Published:** 2026-10-22

**Authors:** Yousra Ramadan, Marwa Baraka, Jeffrey A. Dean, Nadia A. Wahba

**Affiliations:** 1 Department of Pediatric Dentistry and Dental Public Health, Faculty of Dentistry, Alexandria University, Champolion St., Azarita, Alexandria, 21521 Egypt; 2 Departments of Pediatric Dentistry and Orthodontics, School of Dentistry, Indiana University, Indianapolis, IN USA

**Keywords:** BioFlex crowns, SSCs, Hall technique, Plaque and gingival health

## Abstract

**Background:**

The present study evaluated and compared the clinical and radiographic success of BioFlex crowns (BFCs) versus stainless-steel crowns (SSCs) using Hall Technique (HT) in primary molars.

**Materials and methods:**

This two-arm randomized controlled trial included 74 healthy children aged 4–6 years with ICDAS 3–5 carious mandibular second primary molars. Each child contributed one tooth and was randomly assigned (1:1) to receive BFCs or SSCs using the HT. Baseline examination, radiographs, plaque, and gingival indices were recorded. Crowns were cemented with resin-modified glass ionomer. Primary outcomes were clinical and radiographic success; secondary outcomes included plaque and gingival indices, child and parental satisfaction. Evaluations were performed up to 12 months.

**Results:**

At 12 months, no statistically significant difference in success rates was detected between the groups (BFCs 91.9% vs. SSCs 94.6%; RR = 0.97, 95% CI: 0.86–1.10). SSCs had a higher gingival index at 1 week (MD = − 0.32, 95% CI: −0.56–−0.08). BFCs showed higher plaque at 6 months (MD = 0.30) and 12 months (MD = 0.41). Child satisfaction favored BFCs (MD = − 0.67), with greater parental satisfaction for color (MD = 1.73) and overall satisfaction (MD = 0.41).

**Conclusions:**

BFCs are an esthetic alternative to SSCs when used with the HT. They showed no significant differences in short-term clinical success but higher child and parental satisfaction. However, perforation risk and greater plaque accumulation indicate the need for careful case selection and longer-term evaluation.

Trial registration: The trial was registered in the clinicaltrials.gov registry (# NCT06847646).

## Introduction

In recent years, the management of dental caries in children has shifted from extensive caries removal to approaches that emphasize tooth preservation [1]. Minimally Invasive Caries Management (MICM) supports maintaining pulpal vitality while reducing treatment-related discomfort and anxiety. Among these methods, the Hall Technique (HT) has gained prominence [2]. It involves placing a preformed metal crown over a carious primary molar without local anesthesia, caries removal, or tooth preparation [3]. Its effectiveness relies on creating a tight seal that isolates the lesion from the oral environment, limiting bacterial activity and stopping further caries progression [4]. 

Conventionally, SSCs have been the preferred material for the HT because of their strength and reliable clinical outcomes [5, 6]. However, their metallic appearance often raises aesthetic concerns, particularly among parents seeking more natural-looking restorative options for their children [7]. This limitation has promoted interest in alternative crown materials that provide both functional and aesthetic benefits [8]. 

BioFlex crowns (BFCs) are among the recently introduced esthetic alternatives in pediatric restorative dentistry. They were introduced as tooth-colored crowns fabricated from a hybrid resin polymer material [9]. They are known for their ability to adapt to tooth contours because of their material properties. When placed, they show gentle resistance, called a flex, as they slide over and adapt to the tooth’s natural contours, facilitating crown adaptation during placement [10]. Several studies have reported favorable clinical and radiographic outcomes, including good gingival health, adequate retention, and high parental satisfaction [11–13]. However, most of the available studies have evaluated BFCs within conventional restorative protocols that involve caries removal and tooth preparation. In addition, the existing evidence is limited by relatively small sample sizes and short follow-up periods. Consequently, evidence regarding the overall effectiveness of BFCs remains limited, and their performance when used with minimally invasive approaches such as the HT remains uncertain [14]. Therefore, the present study aims to clinically and radiographically evaluate the effectiveness of BFCs in comparison with SSCs when used with HT for the management of carious lesions in primary molars. The null hypothesis was that there would be no statistically significant differences between the two crown types in terms of clinical and radiographic success, plaque accumulation, gingival health, or child and parental satisfaction over a 12- month period.

## Methods

### Study design and ethical considerations

This study was a two-arm, parallel-group randomized controlled trial with a 1:1 allocation ratio, reported in accordance with CONSORT guidelines [15]. The PICOT question was: Do asymptomatic primary teeth with carious lesions (Population; P) treated with BFCs (Intervention; I), compared with the conventional SSCs (Control; C) using the HT, demonstrate differences in clinical and radiographic success (Outcome; O) over one year (Time: T) [16]?.

Ethical approval was obtained from the Research Ethics Committee of the Faculty of Dentistry, Alexandria University (IRB No. 001056 – IORG 0008839). Participant recruitment and data collection occurred between August 2024 and August 2025. The trial was retrospectively registered at ClinicalTrials.gov (NCT06847646) on February 19, 2025. The complete study protocol is available from the corresponding author upon reasonable request. Because the trial was registered retrospectively, the registry record did not fully specify certain methodological details, including the restriction of recruitment to mandibular second primary molars and the assessment schedule for individual outcomes, although these aspects were predefined in the original study protocol and remained unchanged throughout the study. The registered protocol included additional outcomes, such as temporomandibular joint assessment and occlusal vertical dimension, which are outside the scope of the present report and will be presented in a companion manuscript. The use of BFCs with the HT represents an off-label application. This was clearly disclosed to parents/guardians during the informed consent process before enrolment, including an explanation of the available evidence, expected benefits, and potential risks. Written informed consent was obtained before participation.

### Sample size estimation

The sample size calculation was based on the clinical crown-performance outcome of intact and retentive crowns at 12 months. The present study was designed as a comparative superiority trial to detect a difference between BFCs and SSCs, rather than as a formal non-inferiority or equivalence trial; therefore, no non-inferiority or equivalence margin was prespecified. Patil et al. [11] reported that 96% of SSCs and 89.35% of BFCs were intact and retentive after 12 months, corresponding to an expected absolute difference of 6.65% (95% CI: −2.20% to 28.96%). This difference was considered clinically relevant for detecting a potential difference in crown performance over 12 months. Based on a 95% confidence level, 80% study power, and 5% alpha error, the minimum sample size was calculated as 35 patients per group and increased to 37 per group to allow for potential attrition, giving a total sample size of 74 patients [17]. No formal adjustment was made for co-primary outcomes; radiographic success was included as a co-primary biological/safety outcome, while the sample size was driven by the clinical crown-performance endpoint. The sample size was calculated using the MedCalc Statistical Software version 19.0.5 (MedCalc Software Ltd., Ostend, Belgium).

### Participants eligibility

Participants were recruited from the outpatient clinic of the Pediatric Dentistry and Dental Public Health Department, Faculty of Dentistry, Alexandria University, Egypt, where the clinical trial was also conducted. Children aged 4–6 years, free from systemic diseases, special health care needs, or clinical signs or symptoms of bruxism, and exhibiting cooperative behavior (positive or definitely positive) according to Frankl’s Behavior Rating Scale [18], were included if they had one asymptomatic mandibular second primary molar with occlusal or occluso-proximal caries extending into dentine, corresponding to ICDAS codes 4–5, or ICDAS code 3 only when radiographic assessment confirmed dentine involvement [19], provided that a sufficient band of sound dentine was present between the carious lesion and the pulp, as confirmed clinically and radiographically, to ensure the absence of pulpal involvement. In addition, lesions were required to be active, and patients were assessed as having moderate to high caries risk, supporting the indication for full coverage restoration. Each child contributed only one eligible tooth, and participants were randomly assigned (1:1) to receive either BFCs or SSCs using the HT. Teeth were excluded if they presented with clinical or radiographic evidence of spontaneous pain, pulp exposure, pulpitis, fistulous tract, and/or abscess formation, inter-radicular radiolucency, furcation involvement. Moreover, teeth exhibiting pathological mobility due to root resorption, assessed by gently placing the tips of tweezers in the occlusal fossa and rocking the tooth in a buccolingual direction, were also excluded [7]. 

### Randomization technique, allocation concealment, grouping, and blinding

Participants who met the inclusion criteria were randomly assigned, using a computer-generated randomization list, to one of two arms based on the type of crown used: Group I (study group), in which children received BFCs using the HT (*n* = 37), or Group II (control group), in which children received SSCs using the HT (*n* = 37). Allocation was performed by an independent individual not involved in the trial, using block randomization with a fixed block size of 4 to ensure equal group sizes and an equal allocation ratio. Allocation concealment was ensured using sequentially numbered, opaque, sealed envelopes containing identical sheets of paper indicating the assigned treatment group. The envelopes were prepared and securely kept by a separate independent third party who was not involved in participant recruitment, allocation, treatment, or outcome assessment. Each envelope was opened only at the time of treatment after the participant had been enrolled in the study. While the operator was initially blinded to the participants’ group allocation during the randomization process, blinding of the operator and participants during the clinical procedure was not possible due to the clear differences in appearance and handling between BFCs and SSCs [20]. 

### Intra-examiner calibration

Prior to study initiation, the principal investigator underwent training and calibration to standardize the clinical and radiographic evaluation procedures. This training included review of the diagnostic and evaluation criteria for all study outcomes, namely clinical success, radiographic success, plaque index, and gingival index.

To assess intra-examiner reliability, 20% of an additional sample of children with similar clinical conditions, who were not included in the main trial sample, were examined twice by the same investigator with a two-week interval between assessments. Agreement between the two assessments was calculated using Cohen’s kappa coefficient [21]. The intra-examiner kappa values were 0.88 for clinical success assessment, 0.85 for radiographic evaluation, 0.90 for plaque index, and 0.87 for gingival index, indicating strong agreement.

To further enhance the reliability of radiographic assessment, a random subset of radiographs (*n* = 10) was independently reassessed by another examiner, demonstrating 100% inter-examiner agreement (kappa = 1.00).

### Pilot study

A six-month pilot study was initially conducted with three treatment groups: HT using SSCs, HT using BFCs, and a modified HT, in which occlusal reduction was performed before crown placement [22]. The primary objective of this preliminary phase was to assess the feasibility of crown placement, evaluate the performance of the different methods applied, and refine clinical procedures before initiating the main randomized controlled trial. Based on a desired statistical power of 90% and a two-sided significance level of 5%, a sample size of five participants per treatment arm was calculated, assuming a large standardized effect size of 0.8. During placement of the first BFC using the modified HT, an open margin was detected radiographically. The crown was subsequently removed, a full preparation was performed, and the crown was re-cemented. In light of this finding, the modified approach was considered clinically unsuitable and was excluded from the main trial. Consequently, only the true HT (without tooth preparation) was employed for both SSCs and BFCs in the randomized controlled trial.

### Intervention

#### Initial clinical procedures and patient preparation

Patients underwent a preoperative clinical examination using ICDAS II scoring system [23] to determine the severity and activity of carious lesions. Preoperative digital periapical radiographs were taken using a paralleling device (Rinn XCP, Dentsply Sirona, Charlotte, NC, USA) to ensure precise and reproducible alignment of the dental film and (E-Speed intraoral film, Kodak, Rochester, NY, USA) the X-ray tube (Planmeca ProX, Planmeca, Helsinki, Finland; parameters: 60 kVp, 7 mA, 0.2 s exposure). During the first visit, caries risk was assessed, and oral hygiene instructions were provided and demonstrated, including proper brushing twice daily with a standard fluoridated toothpaste appropriate for their age (1000–1450 ppm fluoride) and flossing when needed. Teeth were then professionally cleaned to remove supra- and subgingival deposits, preoperative plaque and gingival indices were recorded, and topical fluoride (5% sodium fluoride varnish (Duraphat), Colgate-Palmolive, New York, NY, USA) was applied. When required, orthodontic separators (Dentaurum elastomeric separators, Dentaurum, Ispringen, Germany) were placed to create adequate space for crown fitting, and a second appointment was scheduled in 3–5 days.

#### Crown placement procedure

At the second visit, the orthodontic separators were carefully removed using an excavator. Any loose plaque or food debris was gently cleaned from the cavity with wet cotton pellets and an excavator, without removing any caries. Children in Group I (study group) received BFCs (NuSmile, Houston, US), while those in Group II (control group) received conventional SSCs (3 M™ ESPE™, St. Paul, MN, USA). The correct crown size was selected to cover all cusps and reach the contact points, ensuring a slight “spring-back” sensation when seated along the gingival margin.

The tooth was isolated with cotton rolls, and the internal surface of the crown was dried with cotton pellets. Resin-modified glass ionomer luting cement (RMGIC) (FujiCem 2, GC Corporation, Tokyo, Japan) was mixed for 10 s and filled to about two-thirds of the crown, avoiding air bubbles. The same cement was used identically in both study groups. The crown was then positioned and seated with firm finger pressure, and the child was instructed to bite gently to ensure full adaptation. Once properly seated, excess cement was removed, interproximal contacts were flossed, and the child was asked to continue biting on a cotton roll for an additional 2–3 min to allow the cement to set completely.

#### Outcome assessment

**Fig. 1:**
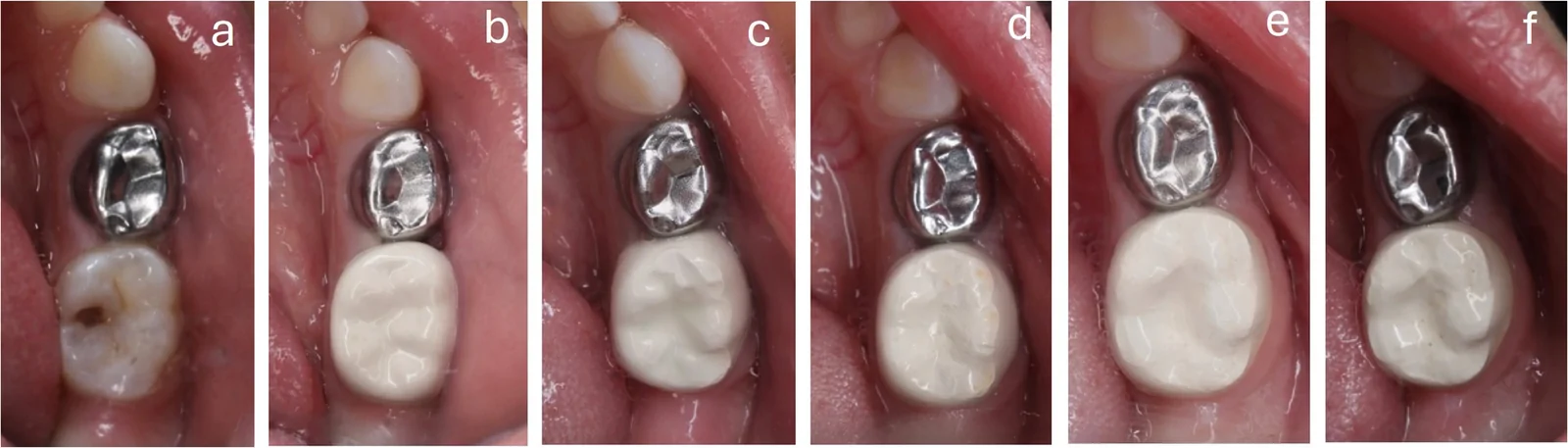
Shows the clinical evaluation of BFC on tooth 85 with an ICDAS score of 5 over 12 months. (**a**) preoperative, (**b**) immediate postoperative, (**c**) 1 month postoperative, (**d**) 3 months postoperative, (**e**) 6 months postoperative, and (**f**) 12 months postoperative. Clinical success was assessed according to the criteria described by Innes et al. [31] , and the crown was rated successful. Progressive occlusal wear of BFC can be observed during the follow-up period

**Fig. 2:**
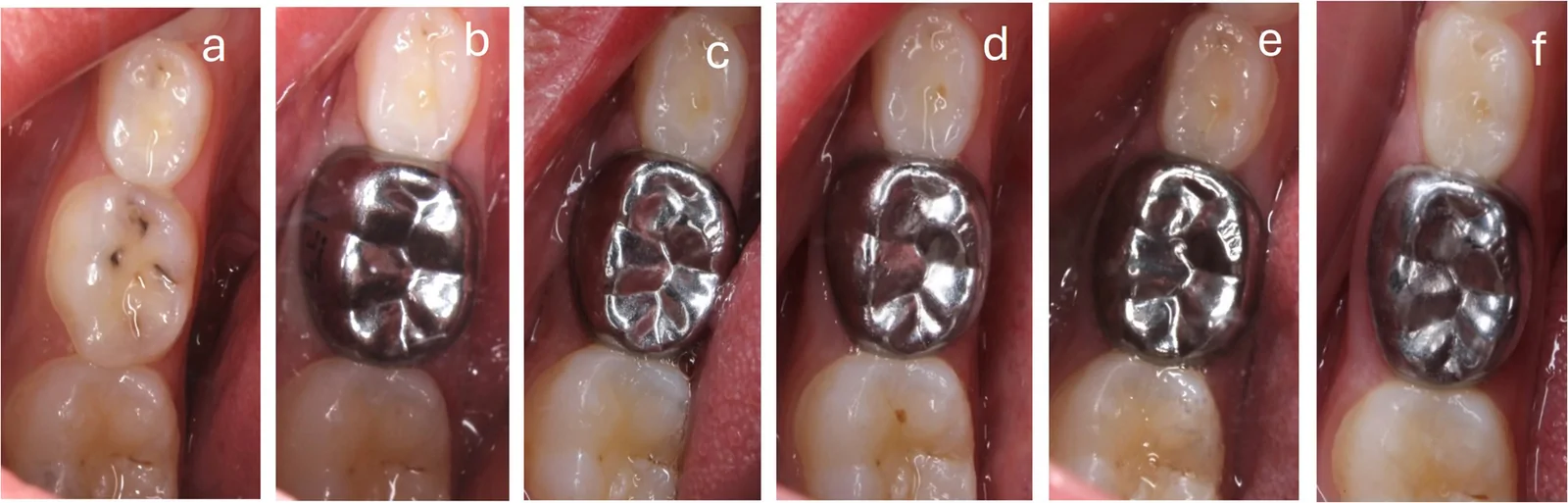
Shows the clinical evaluation of SSC on tooth number 75 with an ICDAS score of 4 over 12 months. (**a**) preoperative, (**b**) immediate postoperative, (**c**) 1 month postoperative, (**d**) 3 months postoperative, (**e**) 6 months postoperative, and (**f**) 12 months postoperative. The crown remained clinically successful throughout the observation period.

**Fig. 3:**
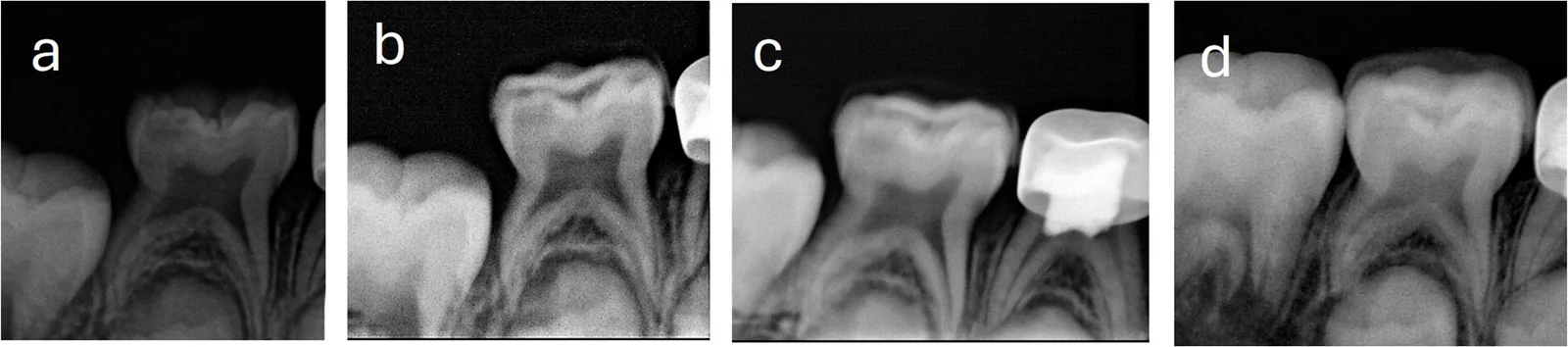
Shows periapical x-rays of BFC on tooth 75 over 12 months. (**a**) preoperative, (**b**) immediate postoperative, (**c**) 6 months postoperative, and (**d**) 12 months postoperative. The crown demonstrated successful radiographic performance, with no evidence of radicular radiolucency, internal or external root resorption, or widening of the periodontal ligament space observed throughout the follow-up period

**Fig. 4:**
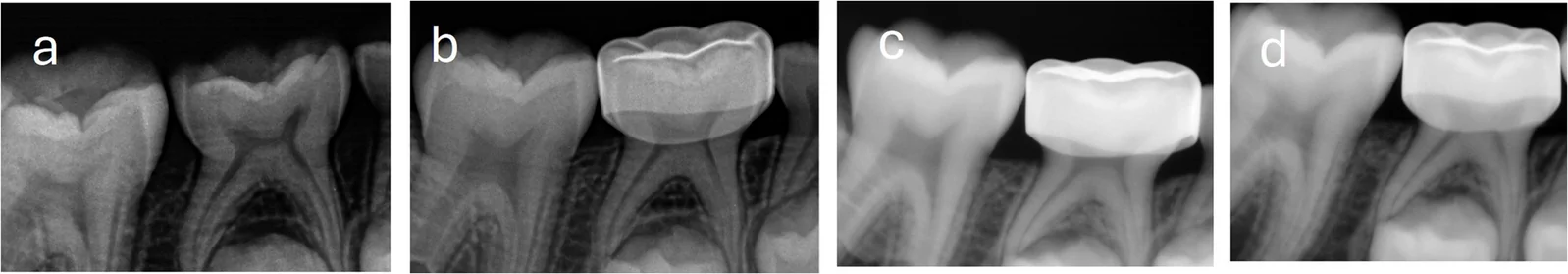
Shows periapical x-rays of SSC on tooth 75 over 12 months. (**a**) preoperative, (**b**) immediate postoperative, (**c**) 6 months postoperative, and (**d**) 12 months postoperative. The crown’s overall radiographic performance was rated successful, with no signs of periapical pathology, root resorption, or periodontal ligament widening

Co-primary outcomes were clinical and radiographic success of both crowns. Clinical success, according to the criteria described by Innes et al. [6] was defined as the absence of clinical signs or symptoms of failure (e.g., pain, swelling, sinus tract, abnormal mobility, tenderness to percussion, or restoration failure). Crown loss or perforation was classified as a minor failure, while irreversible pulpitis, fistula, or abscess requiring pulp therapy or extraction constituted a major failure (Figs. 1 and 2). This outcome was recorded as a dichotomous variable (success/failure), assessed at each follow-up visit (1 week, 1, 3, 6, and 12 months), and summarized as the number and percentage of successful cases per group. Radiographic success was defined as the absence of radicular radiolucency, internal or external root resorption, or periodontal ligament space widening [24]. Radiographic evaluations were performed only for teeth that remained clinically intact at each follow-up visit. Teeth that experienced clinical failure, such as crown loss or perforation, were classified as treatment failures and were not subjected to further radiographic assessment. This outcome was recorded as a dichotomous variable (success/failure), assessed at 6 and 12 months, and summarized as the number and percentage of successful cases per group (Figs. 3 and 4).

Secondary outcomes encompassed plaque accumulation, gingival health, as well as child and parent/guardian satisfaction. Plaque accumulation on the four crown surfaces was assessed using the Silness and Löe Plaque Index [25], and were scored from 0 (no plaque) to 3 (abundant plaque). The sum of the four scores was divided by 4 to obtain the final score. Plaque index was analyzed as an ordinal/continuous variable and summarized using mean ± SD and median (IQR) at each timepoint.

Gingival health was evaluated using the Löe and Silness index [26], with scores of 0 (no inflammation), 1 (mild inflammation), 2 (moderate inflammation), and 3 (severe inflammation). Gingival index was analyzed as an ordinal/continuous variable and summarized using mean ± SD and median (IQR) at each follow-up visit.

Crown performance and immediate postoperative child satisfaction were recorded after cementation using the Facial Image Scale (FIS), a modified version of the Maunuksela et al. scale [27], and analyzed as an ordinal outcome.

Parental satisfaction regarding crown size, shape, color, retention, recommendation, and overall satisfaction was evaluated using a modified structured questionnaire adopted from previously published and validated instruments in pediatric dental studies assessing esthetic crowns in primary teeth. The satisfaction questionnaire used in the present study differed slightly from that described in the trial registration; specifically, the item related to durability was omitted because restoration durability was assessed objectively through the clinical evaluation of restoration success. The questionnaire consisted of 6 items scored on a 5-point Likert scale ranging from “very dissatisfied” to “very satisfied.” [28, 29] Responses were summarized as mean scores per domain and overall satisfaction.

The questionnaire was administered by the principal investigator after crown placement during the follow-up visit, once parents had the opportunity to evaluate the restoration. Parents completed the questionnaire independently without influence from the clinical operator. Responses were collected anonymously, and parents were informed that their answers would be used solely for research purposes to minimize social desirability bias.

Follow-up evaluations were at 1 week, 1, 3-, 6-, and 12-months post-cementation. Postoperative periapical radiographs were taken using a paralleling device immediately after crown placement and again at 6, and 12 months (Figs. 3 and 4) [30]. 

#### Adverse events assessment

Adverse events were monitored throughout the study period in accordance with CONSORT recommendations. At each follow-up visit, participants were clinically examined for potential harms or unintended effects, including postoperative pain, soft tissue irritation, cement-related complications, and crown failures requiring retreatment. Any reported symptoms or clinical signs were recorded. In cases where crown failure occurred, the failed crown was recorded as a failure, removed, and replaced with a new crown using the same clinical protocol.

### Statistical analysis

Descriptive statistics were presented as frequencies and percentages for categorical variables, and as means, standard deviations (SD), medians, and interquartile ranges (IQR) for continuous and ordinal variables. Normality was assessed using descriptive statistics, graphical methods, and Shapiro Wilk normality test, which indicated non-normal data distribution. For binary outcomes (clinical and radiographic success), comparisons between groups at each timepoint were performed using Fisher’s exact test. In addition to p values, relative risks (RRs) with 95% confidence intervals (CIs) were calculated. For continuous and ordinal outcomes (plaque index, gingival index, child satisfaction, and parental satisfaction), between-group comparisons at each timepoint were performed using the Mann–Whitney U test. Mean differences with 95% confidence intervals were calculated. Changes across time within each group were analyzed using the Friedman test for quantitative and ordinal data and Cochran’s Q test for nominal data. When statistically significant, Bonferroni-adjusted post-hoc pairwise comparisons were performed. The analysis was conducted according to the intention-to-treat principle, with all 74 randomized participants analyzed according to their original treatment allocation. No participants were lost to follow-up during the 12-month study period; therefore, there were no missing outcome data attributable to participant attrition. For binary clinical outcomes, when a clinical failure occurred at a given follow-up visit, the affected restoration was classified as a failure at all subsequent follow-up visits. This procedure represents a failure-carried-forward approach and was used to maintain the clinical failure status of the restoration throughout the remaining follow-up period. Thus, failure carried forward was not used to impute missing observations due to loss to follow-up, but rather to account for the persistence of a documented clinical failure at subsequent assessments. A p value < 0.05 was considered statistically significant. Data were analyzed using IBM SPSS Statistics for Windows, version 26.0 (IBM Corp., Armonk, NY, USA).

## Results

**Fig. 5:**
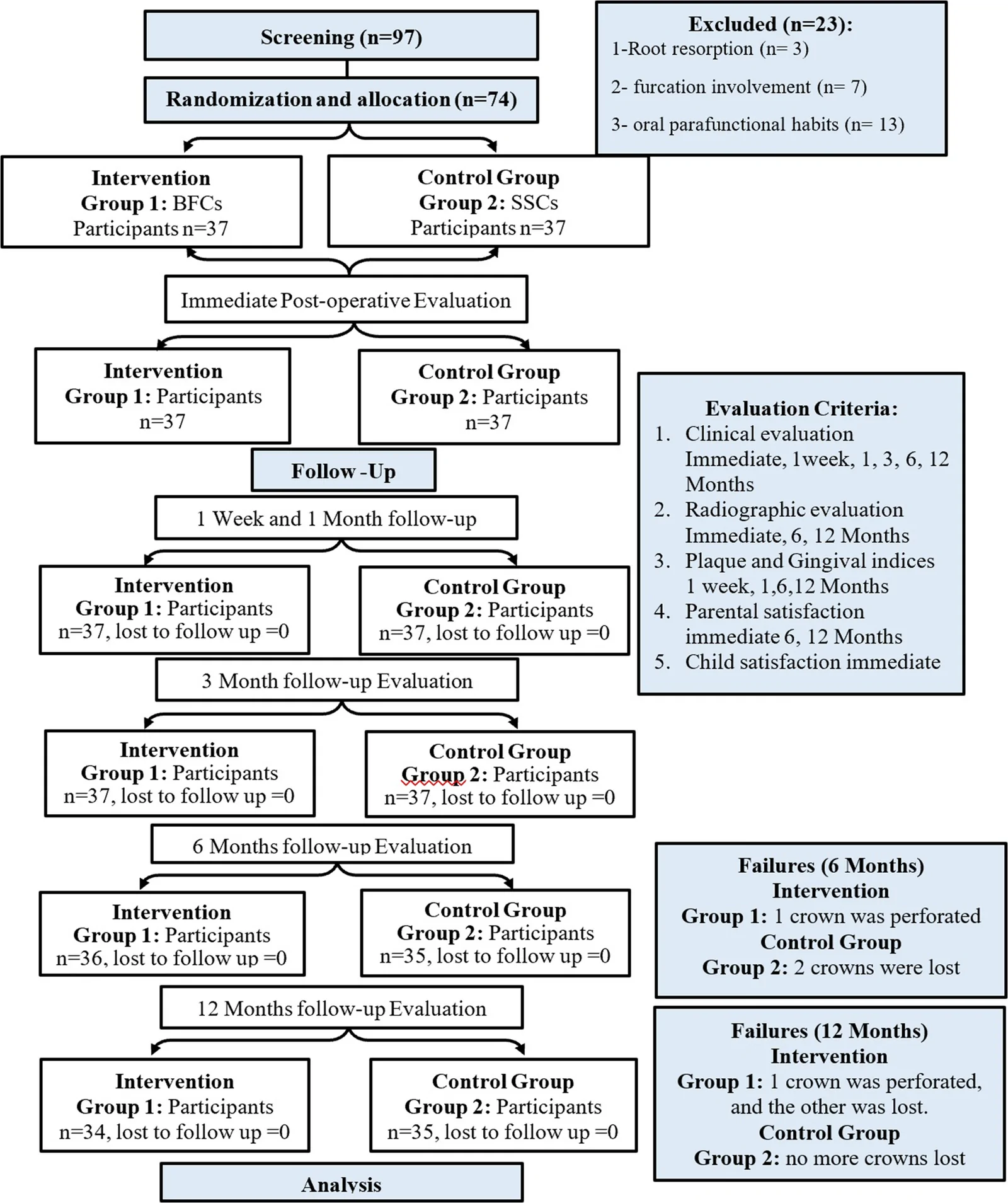
Consort flow chart

A total of 74 children were recruited and randomly allocated into two groups: 37 in the BFC group and 37 in the SSC group. All participants underwent the preoperative assessment. The demographic and baseline characteristics of the participants are presented in Table 1. No statistically significant differences were observed between the study groups regarding age, gender, tooth side, lesion type, lesion severity (ICDAS score), and the use of orthodontic separators (*p* = 0.62, *p* = 0.81, *p* = 0.77, *p* = 0.69, *p* = 0.84, and *p* = 0.65, respectively). (Fig. 5).

**Table 1:** Demographic data of the participating children among the study groups

Variable			BFCs (*n* = 37)	SSCs (*n* = 37)	*p*-value
Child-level variables	Age in years (Mean ± SD)		5.2 ± 0.8	5.3 ± 0.7	0.62
	Gender, n (%)	Male	19 (51.4)	21 (56.7)	0.81
		Female	18 (48.6)	16 (43.2)	
Tooth-level variables	Side	Right	20 (54.1)	21 (56.8)	0.77
		Left	17 (45.9)	16 (43.2)	
	Lesion type	Occlusal	15 (40.5)	14 (37.8)	0.69
		Occlusoproximal	22 (59.5)	23 (62.2)	
	Lesion severity (ICDAS score)	Code 3	10 (27.0)	9 (24.3)	0.84
		Code 4	13 (35.1)	14 (37.8)	
		Code 5	14 (37.9)	14 (37.9)	
	Separator used	Yes	24 (64.9)	26 (70.3)	0.65
		No	13 (35.1)	11 (29.7)	

*SD*: Standard Deviation

P value: Group comparisons were performed using independent t-test for continuous variables and chi-square test for categorical variables

### Clinical evaluation

**Fig. 6:**
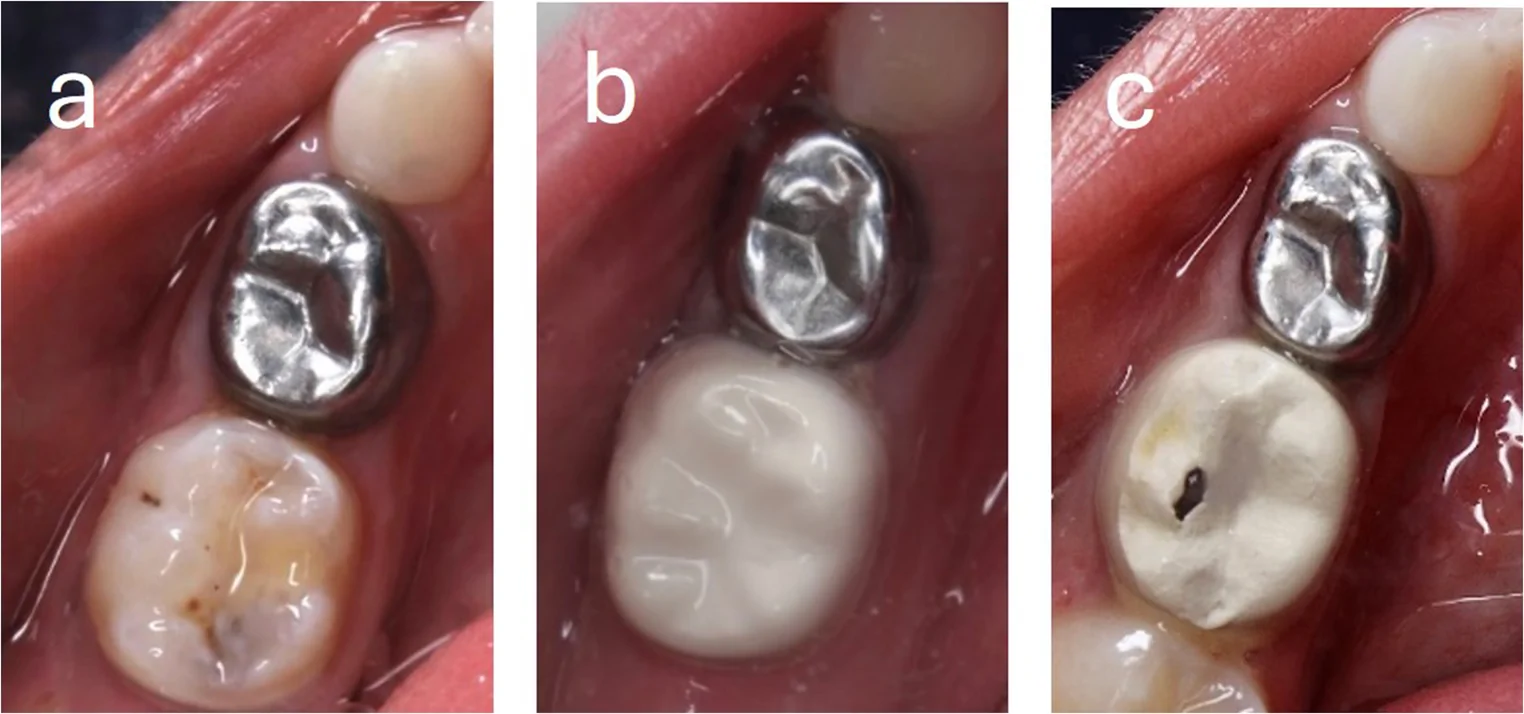
Shows clinical evaluation of a BFC on tooth 75 with an ICDAS score of 4 at different time points (**a**) preoperative, (**b**) immediately postoperative. (**c**) shows a notable perforation on BFC after 6 months

Table 2 summarizes the clinical success rates of BFCs and SSCs over the 12-month follow-up period. Both groups demonstrated favourable clinical performance, with no statistically significant differences at any time point. Both crowns had 100% success at 3 months, followed by a slight decline at 6 months (97.3% for BFCs and 94.6% for SSCs) (RR = 1.03; 95% CI: 0.94–1.13; *p* = 0.99). By 12 months, success remained high (91.9% for BFCs vs. 94.6% for SSCs), with an RR of 0.97 (95% CI: 0.86–1.10; *p* = 0.99). Minor failures were infrequent. Crown perforation occurred exclusively in the BFC group at 6 months (2.7%) and 12 months (5.4%), with RR values of 3.00 (95% CI: 0.13–71.35; *p* = 0.62) and 5.00 (95% CI: 0.25–100.72; *p* = 0.49), respectively, indicating no significant increased risk compared to SSCs (Fig. 6). Crown loss occurred in two SSC cases at both 6 and 12 months (5.4%), and in one BFC case (2.7%) at 12 months only, with no statistically significant difference between groups at either timepoint (RR = 0.20, 95% CI: 0.01–4.03, *p* = 0.49 at 6 months; RR = 0.50, 95% CI: 0.05–5.28, *p* = 0.62 at 12 months). No major failures were observed in either group throughout the study period (*p* = 1.00).

**Table 2:** Comparison of clinical success between the two study groups across time

			BioFlex	SSC	RR (95% CI)	P value 1
			n (%)			
Success		**1 week**	37/37 (100%)	37/37 (100%)	-	1.00
		**1 month**	37/37 (100%)	37/37 (100%)	-	1.00
		**3 months**	37/37 (100%)	37/37 (100%)	-	1.00
		**6 months**	36/37 (97.3%)	35/37 (94.6%)	1.03 (0.94, 1.13)	0.99
		**12 months**	34/37 (91.9%)	35/37 (94.6%)	0.97 (0.86, 1.10)	0.99
		**P value 2**	0.58	0.17		
Minor failure	**Crown perforation**	**1 week**	0/37 (0%)	0/37 (0%)	-	1.00
		**1 month**	0/37 (0%)	0/37 (0%)	-	1.00
		**3 months**	0/37 (0%)	0/37 (0%)	-	1.00
		**6 months**	1/37 (2.7%)	0/37 (0%)	3.00 (0.13–71.35)	0.62
		**12 months**	2/37 (5.4%)	0/37 (0%)	5.00 (0.25–100.72)	0.49
		**P value 2**	0.17	1.00		
	**Crown loss**	**1 week**	0/37 (0%)	0/37 (0%)	-	1.00
		**1 month**	0/37 (0%)	0/37 (0%)	-	1.00
		**3 months**	0/37 (0%)	0/37 (0%)	-	1.00
		**6 months**	0/37 (0%)	2/37 (5.4%)	0.20 (0.01–4.03)	0.49
		**12 months**	1/37 (2.7%)	2/37 (5.4%)	0.50 (0.05–5.28)	0.62
		**P value 2**	0.41	0.10		
Major failure		**1 week**	0/37 (0%)	0/37 (0%)	-	1.00
		**1 month**	0/37 (0%)	0/37 (0%)	-	1.00
		**3 months**	0/37 (0%)	0/37 (0%)	-	1.00
		**6 months**	0/37 (0%)	0/37 (0%)	-	1.00
		**12 months**	0/37 (0%)	0/37 (0%)	-	1.00
		**P value 2**	1.00	1.00		

n: frequency, %: percentage, RR: Relative Risk, CI: Confidence Interval

P value 1: Comparisons between the two groups using Fisher exact test

P value 2: Comparisons between different timepoints using Cochran’s Q test

### Radiographic evaluation

Table 3 shows that radiographic success rates were high for both groups. Radiographic outcomes were assessed only for teeth that remained clinically intact, as teeth that failed clinically (e.g., crown loss or perforation) were not evaluated radiographically. There were no statistically significant differences at any time point: 97.3% for BFCs compared to 94.6% for SSCs at 6 months (RR = 1.03; 95% CI: 0.94–1.13; *p* = 0.99), indicating a minimal and non-significant difference in favor of BFCs, with the confidence interval including 1. At 12 months, radiographic success was 91.9% for BFCs and 94.6% for SSCs (RR = 0.97; 95% CI: 0.86–1.10; *p* = 0.99). Moreover, no significant changes within either group were detected over time (BFCs *p* = 0.60; SSCs *p* = 0.22). The small decline in radiographic success over time was entirely attributable to clinical failures, which were classified as failures in the overall success rate regardless of radiographic findings.

**Table 3:** Comparison of radiographic success between the two study groups across time

	BioFlex	SSC	RR (95% CI)	*P* value 1
	*n* (%)			
Pre-operative	37/37 (100%)	37/37 (100%)	-	1.00
6 months	36/37 (97.3%)	35/37 (94.6%)	1.03 (0.94, 1.13)	0.99
12 months	34/37 (91.9%)	35/37 (94.6%)	0.97 (0.86, 1.10)	0.99
P value 2	0.60	0.22		

n: frequency, %: percentage, RR: Relative Risk, CI: Confidence Interval

P value 1: Comparisons between the two groups using Fisher exact test

P value 2: Comparisons between different timepoints using Cochran’s Q test

### Gingival index

**Fig. 7:**
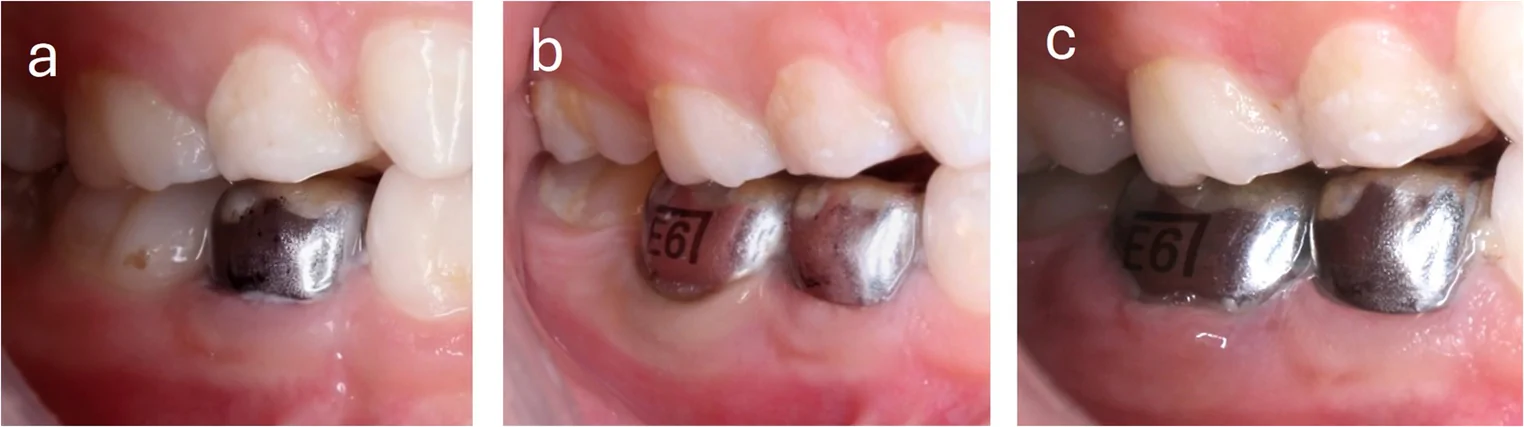
Shows the gingival tissue response after SSC placement on tooth 85 after 1 week, (**a**) preoperative, (**b**) immediately postoperative, (**c**) 1 week postoperative, with marked gingival inflammation around SSC

At the preoperative assessment, gingival index scores were comparably low for both BFCs and SSCs (mean difference = − 0.03; 95% CI: −0.15– 0.09; *p* = 0.65), indicating no significant baseline difference. After one week, SSCs demonstrated significantly higher gingival index scores compared to BFCs (0.46 ± 0.65 versus 0.14 ± 0.35; mean difference = − 0.32; 95% CI: −0.56–−0.08; *p* = 0.01) (Fig. 7). No significant differences were detected between groups at one month (mean difference = − 0.19; 95% CI: −0.38–0.00; *p* = 0.10), six months (mean difference = − 0.08; 95% CI: −0.23–0.07; *p* = 0.29), or twelve months (mean difference = − 0.11; 95% CI: −0.28–0.06; *p* = 0.21). Changes within each group over time were not statistically significant for either BFCs (*p* = 0.67) or SSCs (*p* = 0.10) (Table 4).

**Table 4:** Comparison of gingival index between the two study groups across time

		BioFlex	SSC	Mean difference (95% CI)	*P* value 1
Pre-operative	**Mean (SD)**	0.05 (0.23)	0.08 (0.28)	−0.03 (− 0.15, 0.09)	0.65
	**Median (IQR)**	0.00 (0.00, 0.00)	0.00 (0.00, 0.00)		
1 week	**Mean (SD)**	0.14 (0.35)	0.46 (0.65)	−0.32 (− 0.56, − 0.08)	0.01*
	**Median (IQR)**	0.00 (0.00, 0.00)	0.00 (0.00, 1.00)		
1 month	**Mean (SD)**	0.08 (0.28)	0.27 (0.51)	−0.19 (− 0.38, 0.00001)	0.10
	**Median (IQR)**	0.00 (0.00, 0.00)	0.00 (0.00, 0.50)		
6 months	**Mean (SD)**	0.08 (0.28)	0.16 (0.37)	−0.08 (− 0.23, 0.07)	0.29
	**Median (IQR)**	0.00 (0.00, 0.00)	0.00 (0.00, 0.00)		
12 months	**Mean (SD)**	0.11 (0.31)	0.22 (0.42)	−0.11 (− 0.28, 0.06)	0.21
	**Median (IQR)**	0.00 (0.00, 0.00)	0.00 (0.00, 0.00)		
P value 2		0.67	0.10		

SD: Standard Deviation, IQR: Interquartile Range, CI: Confidence Interval

P value 1: Comparisons between the two groups using Mann-Whitney U test

P value 2: Comparisons between different timepoints within each group using Friedman test

*statistically significant at p-value < 0.05

### Plaque index

Table 5 presents the comparison of plaque index scores between the two groups across all follow-up periods. No significant differences were detected preoperatively (*p* = 0.69), 1 week (*p* = 1.00), or 1 month (*p* = 0.28). The mean differences between the BFC and SSC groups at these time points were small and the 95% confidence intervals crossed zero (0.03, 95% CI: −0.11–0.17; 0.00, 95% CI: −0.21–0.21; and 0.11, 95% CI: −0.09– 0.31, respectively). However, at 6 months (*p* = 0.04), a significant difference appeared, with a mean difference of 0.30 (95% CI: 0.03–0.57), indicating higher plaque scores in the BFC group. This difference became more pronounced at the 12-month follow-up (*p* = 0.005), where the mean difference increased to 0.41 (95% CI: 0.12–0.70). Within-group comparison also revealed a significant increase in plaque index over time in the BFC group (*p* = 0.002), while the SSC group did not show a significant change (*p* = 0.53).

**Table 5:** Comparison of plaque index between the two study groups across time

		BioFlex	SSC	Mean difference (95% CI)	*P* value 1
Pre-operative	**Mean (SD)**	0.11 (0.35) **a**	0.08 (0.28)	0.03 (− 0.11 to 0.17)	0.69
	**Median (IQR)**	0.00 (0.00, 0.00)	0.00 (0.00, 0.00)		
1 week	**Mean (SD)**	0.27 (0.45) **a**	0.27 (0.45)	0.00 (− 0.21 to 0.21)	1.00
	**Median (IQR)**	0.00 (0.00, 1.00)	0.00 (0.00, 1.00)		
1 month	**Mean (SD)**	0.30 (0.46) **a**	0.19 (0.40)	0.11 (− 0.09 to 0.31)	0.28
	**Median (IQR)**	0.00 (0.00, 1.00)	0.00 (0.00, 0.00)		
6 months	**Mean (SD)**	0.54 (0.69) **b**	0.24 (0.49)	0.30 (0.03 to 0.57)	0.04*
	**Median (IQR)**	0.00 (0.00, 1.00)	0.00 (0.00, 0.00)		
12 months	**Mean (SD)**	0.68 (0.71) **b**	0.27 (0.56)	0.41 (0.12 to 0.70)	0.005*
	**Median (IQR)**	1.00 (0.00, 1.00)	0.00 (0.00, 0.00)		
P value 2		0.002*	0.53		

*SD*: Standard Deviation, *IQR*: Interquartile Range, *CI*: Confidence Interval

P value 1: Comparisons between the two groups using Mann-Whitney U test

P value 2: Comparisons between different timepoints within each group using Friedman test

*statistically significant at p-value < 0.05

a-b: different letters denote significant differences between timepoints using Bonferroni correction

### Child satisfaction

Child satisfaction was significantly higher with BFCs than SSCs (*p* = 0.001). 48.6% of BFCs’ children reported very happy responses, compared with 13.5% of SSCs. Neutral responses were more frequent with SSCs (29.7%) than with BFCs (13.5%), and negative feelings were reported only in the SSC group (8.1%). Mean satisfaction scores also indicated better outcomes for BFCs (1.65 ± 0.72) compared with SSCs (2.32 ± 0.82). with a mean difference of − 0.67 (95% CI: −1.02 − 0.32), indicating significantly better satisfaction with BFCs (Table 6).

**Table 6:** Comparison of child satisfaction between the two study groups immediately post-treatment

	BioFlex	SSC	Mean difference (95% CI)	P value
	N (%)			
Very happy (1)	18 (48.6%)	5 (13.5%)	**-**	0.001*
Happy (2)	14 (37.8%)	18 (48.6%)		
Neutral (3)	5 (13.5%)	11 (29.7%)		
Sad (4)	0 (0%)	3 (8.1%)		
Very sad (5)	0 (0%)	0 (0%)		
Mean (SD)	1.65 (0.72)	2.32 (0.82)	−0.67 (− 1.02 to − 0.32)	
Median (IQR)	2.00 (1.00, 2.00)	2.00 (2.00, 3.00)		

*SD*: Standard Deviation, *IQR*: Interquartile Range, *CI*: Confidence Interval

Mann-Whitney U test was used

*statistically significant at p-value < 0.05

### Parental satisfaction

Parental satisfaction, illustrated in Table 7, was higher with BFCs than with SSCs across all follow-up assessments, particularly for color (postoperative mean difference 1.73, 95% CI: 1.32–2.14; 6 months 1.06, 95% CI: 0.70–1.42; 12 months 1.06, 95% CI: 0.71–1.41; *p* < 0.001) and overall satisfaction (postoperative 0.41, 95% CI: 0.17–0.65; 6 months 0.40, 95% CI: 0.17–0.63; 12 months 0.35, 95% CI: 0.13–0.57; *p* = 0.01). Satisfaction with shape was significantly greater for BFCs at 6 months (mean difference 0.54, 95% CI: 0.36–0.72; *p* < 0.001). No significant differences were observed for size, retention, or recommendation, as their confidence intervals included zero.

**Table 7:** Comparison of parental satisfaction between the two study groups

			BioFlex	SSC	Mean difference (95% CI)	P value 1
Color	**Post-operative**	**Mean (SD)**	4.54 (0.61)	2.81 (1.05) **a**	1.73 (1.32 to 2.14)	< 0.001*
		**Median (IQR)**	5.00 (4.00, 5.00)	3.00 (2.00, 3.50)		
	**6 months**	**Mean (SD)**	4.03 (0.65)	2.97 (0.89) **b**	1.06 (0.70 to 1.42)	< 0.001*
		**Median (IQR)**	4.00 (4.00, 4.00)	3.00 (2.00, 3.00)		
	**12 months**	**Mean (SD)**	4.03 (0.66)	2.97 (0.76) **b**	1.06 (0.71 to 1.41)	< 0.001*
		**Median (IQR)**	4.00 (4.00, 4.00)	3.00 (2.00, 3.00)		
	**P value 2**		0.10	0.001*		
Shape	**Post-operative**	**Mean (SD)**	4.84 (0.37)	5.00 (0.00) **a**	−0.16 (− 0.30 to − 0.02)	0.10
		**Median (IQR)**	5.00 (5.00, 5.00)	5.00 (5.00, 5.00)		
	**6 months**	**Mean (SD)**	4.97 (0.17)	4.43 (0.50) **b**	0.54 (0.36 to 0.72)	< 0.001*
		**Median (IQR)**	5.00 (5.00, 5.00)	4.00 (4.00, 5.00)		
	**12 months**	**Mean (SD)**	4.94 (0.24)	5.00 (0.00) **a**	−0.06 (− 0.16 to 0.04)	0.16
		**Median (IQR)**	5.00 (5.00, 5.00)	5.00 (5.00, 5.00)		
	**P value 2**		0.16	< 0.001*		
Size	**Post-operative**	**Mean (SD)**	5.00 (0.00)	5.00 (0.00)	-	1.00
		**Median (IQR)**	5.00 (5.00, 5.00)	5.00 (5.00, 5.00)		
	**6 months**	**Mean (SD)**	5.00 (0.00)	5.00 (0.00)	-	1.00
		**Median (IQR)**	5.00 (5.00, 5.00)	5.00 (5.00, 5.00)		
	**12 months**	**Mean (SD)**	5.00 (0.00)	5.00 (0.00)	-	1.00
		**Median (IQR)**	5.00 (5.00, 5.00)	5.00 (5.00, 5.00)		
	**P value 2**		1.00	1.00		
Overall satisfaction	**Post-operative**	**Mean (SD)**	5.00 (0.00)	4.59 (0.50)	0.41 (0.17 to 0.65)	0.01*
		**Median (IQR)**	5.00 (5.00, 5.00)	5.00 (4.00, 5.00)		
	**6 months**	**Mean (SD)**	4.97 (0.17)	4.57 (0.50)	0.40 (0.17 to 0.63)	0.01*
		**Median (IQR)**	5.00 (5.00, 5.00)	5.00 (4.00, 5.00)		
	**12 months**	**Mean (SD)**	4.97 (0.17)	4.62 (0.49)	0.35 (0.13 to 0.57)	0.01*
		**Median (IQR)**	5.00 (5.00, 5.00)	5.00 (4.00, 5.00)		
	**P value 2**		0.37	0.88		
Retention	**Post-operative**	**Mean (SD)**	5.00 (0.00)	5.00 (0.00)	-	1.00
		**Median (IQR)**	5.00 (5.00, 5.00)	5.00 (5.00, 5.00)		
	**6 months**	**Mean (SD)**	5.00 (0.00)	5.00 (0.00)	-	1.00
		**Median (IQR)**	5.00 (5.00, 5.00)	5.00 (5.00, 5.00)		
	**12 months**	**Mean (SD)**	5.00 (0.00)	4.97 (0.17)	0.03 (− 0.03 to 0.09)	0.31
		**Median (IQR)**	5.00 (5.00, 5.00)	5.00 (5.00, 5.00)		
	**P value 2**		1.00	0.37		
Recommendation	**Post-operative**	**Mean (SD)**	4.89 (0.32)	4.03 (0.48)	0.86 (0.66 to 1.06)	0.01*
		**Median (IQR)**	5.00 (5.00, 5.00)	5.00 (4.00, 5.00)		
	**6 months**	**Mean (SD)**	4.89 (0.32)	4.77 (0.43)	0.12 (− 0.05 to 0.29)	0.19
		**Median (IQR)**	5.00 (5.00, 5.00)	5.00 (5.00, 5.00)		
	**12 months**	**Mean (SD)**	4.89 (0.32)	4.85 (0.36)	0.04 (− 0.11 to 0.19)	0.69
		**Median (IQR)**	5.00 (5.00, 5.00)	5.00 (5.00, 5.00)		
	**P value 2**		1.00	0.24		

*SD*: Standard Deviation, *IQR*: Interquartile Range, *CI*: Confidence Interval

P value 1: Comparisons between the two groups using Mann-Whitney U test

P value 2: Comparisons between different timepoints within each group using Friedman test

*statistically significant at p-value < 0.05

a-b: different letters denote significant differences between timepoints using Bonferroni correction

## Discussion

As contemporary pediatric dentistry increasingly seeks evidence-based, minimally invasive restorative solutions for primary teeth, BFCs, a tooth-colored, full-coverage restoration, have emerged as a promising esthetic alternative to the conventional SSCs [31]. While SSCs placed using the HT have demonstrated high clinical and radiographic success rates, with up to a 97% survival rate after five years [32], their metallic appearance always remains an esthetic drawback for patients and caregivers [31]. BFCs have helped address this limitation. Despite their potential, clinical trials evaluating the performance of BFCs placed using the HT are lacking. According to the manufacturer’s recommendations, BFCs are not currently indicated for use with the HT; however, this limitation is attributable to insufficient clinical evidence rather than an absolute contraindication. Therefore, their off-label use in the present study was intended to explore their clinical performance under conditions where evidence remains limited. The main aim of this study was to compare the clinical and radiographic success rates of SSCs and BFCs when placed using the HT, thereby combining the improved esthetics of BFCs with the preparation-free advantages of the HT. Up to our knowledge, this study is the first clinical trial to conduct such a comparison using the HT.

The null hypothesis was accepted for the primary outcome, as no statistically significant differences were observed between the two groups neither in clinical nor radiographic success. However, it was partially rejected for the secondary outcomes, as significant differences were found in plaque index scores and in child and parental satisfaction, while gingival index scores did not differ significantly between the groups.

Our study included teeth with ICDAS scores ranging from 3 to 5. While these codes encompass lesions from localized enamel breakdown to cavitated lesions with visible dentine, lesion assessment was based on both clinical and radiographic examination. In cases initially classified as ICDAS 3, inclusion was contingent upon radiographic confirmation of dentine involvement, ensuring appropriate case selection for full-coverage restorations. This approach is consistent with previous studies evaluating the HT, which have included lesions across a range of ICDAS scores (3–5), reflecting variability in clinical thresholds and decision-making. Moreover, the decision to provide full-coverage restorations was not based solely on lesion severity but also on the overall caries risk of the patient and the anticipated prognosis of the tooth. In patients with elevated caries risk or where lesion progression was likely, full-coverage management was considered a more predictable and biologically sound approach to ensure long-term tooth survival [33, 34]. 

Our findings revealed that BFCs and SSCs using HT demonstrated favourable clinical performance throughout the one-year follow-up period, with no notable differences in overall success or minor failures. Both crowns showed complete initial success, with only a slight decrease observed in subsequent evaluations. By the end of the study period, both crown types continued to exhibit high stability and durability. Similar observations were reported by Singh et al., [35] who found that both SSCs and BFCs performed well clinically, with SSCs showing a slightly higher but insignificant difference in retention, although their study did not adopt the HT.

A noteworthy result was the incidence of perforation exclusively in the BFC group at six-and twelve-month follow-up, potentially indicating a material-specific vulnerability to occlusal wear compared to metallic SSCs. While this difference was not statistically significant, the affected patients reported frequent consumption of hard foods such as popcorn, implying that BFCs may be less suitable for patients with high masticatory demands. This observation highlights the importance of patient selection when choosing BFCs, particularly for children with strong bite forces or dietary habits involving hard or abrasive foods. Even in the absence of statistically significant differences, such material-specific implications could affect long-term crown survival and parental satisfaction. Moreover, because the HT relies on maintaining a durable biological seal to arrest caries progression, crown perforation may represent more than a material or esthetic complication. Disruption of the crown integrity could potentially compromise restoration longevity and affect pulpal health over time. Although the present study was not designed to investigate these consequences, the long-term clinical significance of crown perforation remains unknown and requires further evaluation in studies with extended follow-up periods. Our results align with those reported by Patil et al., [11] who observed no early signs of wear for either crown type but noted greater occlusal wear in BFCs at later assessments, including cases where wear occurred both with and without tooth surface exposure, while SSCs showed markedly less wear. In contrast, Metwally et al. [36] found that BFCs exhibited lower wear volumes than SSCs. Notably, neither of these studies employed the HT, yet their short-term reported outcomes parallel our findings, suggesting that the HT does not adversely affect the wear behavior of either crown type.

In our study, both BFCs and SSCs showed similarly healthy gingival conditions at baseline. However, after one week, SSCs showed significantly higher gingival index scores compared to BFCs. This short-term tissue response is in line with the findings of Doostmohammad et al., [37] who reported that the inflammatory biomarker IL-1β, which is a pro-inflammatory cytokine involved in gingival inflammation, increased after one week in the SSC group, more than in the amalgam group, indicating gingival inflammation. They suggested that this early soft tissue inflammation is often due to marginal adaptation or surface roughness and typically subsides as tissues adapt. However, as the follow-up continued, there were no statistically significant differences found between the two crown types. Similarly, Achanta et al., [38] and Singh et al., [35] found no significant difference in the gingival index scores between BFCs and SSCs groups throughout the 1-year follow-up. Abdelhafez and Dhar [31]also reported consistent results, with no significant difference among BFCs, SSCs, and zirconia crowns, although the SSC group had the highest mean gingival index scores among all groups.

During the one-month follow-up period, both BFCs and SSCs showed similarly low plaque accumulation, suggesting that crown material had a minimal initial impact on plaque retention. Over time, however, differences between the two crown types became more apparent. Plaque scores were higher with BFCs. This increase may be attributed to subtle surface changes in BFCs over time or to their specific contours, which can create sites favourable for plaque accumulation. According to Al-Haj et al. [39] the surface roughness of BFCs was significantly higher than that of zirconia crowns. Consequently, plaque accumulation on BFCs may increase over time due to the presence of a relatively rough surface, as smoother crown surfaces are generally more effective in reducing residual food retention, minimizing plaque adhesion, and promoting gingival health [36]. Furthermore, the slightly bulkier cervical profiles of BFCs, designed to compensate for material flexibility, may create plaque-retentive niches along crown margins and proximal contacts, which are more difficult to clean and can facilitate bacterial colonization. These findings are in agreement with those of Ahilya et al. [40] who conducted a split-mouth randomized controlled study, and reported significant differences in plaque scores between teeth restored with SSCs and BFC-restored teeth, favoring SSCs. Conversely, Abdelhafez [31] reported no statistically significant difference in plaque index scores between SSCs, BFCs, and zirconia crowns at different follow-up periods; however, within-group comparisons showed that plaque index scores increased over time in each group, although these changes were not statistically significant.

Importantly, the observed increase in plaque accumulation with BFCs may have clinical implications in the context of the HT, particularly in high-caries-risk children. Sustained plaque retention has been hypothesized to influence gingival health over longer follow-up periods, increase the risk of caries development in adjacent teeth, and affect marginal tissue response around restorations. However, these outcomes were not directly assessed in the present study, and therefore such implications should be interpreted with caution. Further long-term studies are required to determine whether the observed differences in plaque accumulation translate into clinically significant effects on periodontal health or restoration longevity.

In the current study, child satisfaction differed significantly between the two crown types, with BFCs receiving notably higher positive responses than SSCs. Similarly, Rahate et al. [41] reported a higher satisfaction level with BFCs than with SSCs. Parental satisfaction with BFCs was higher, particularly in terms of color, and overall appearance. These findings were consistent with the findings of Al-Haj [9], who reported higher parental satisfaction with BFCs than with SSCs.

Our study has several limitations. The twelve-month follow-up provides insight into short-term performance but may not fully reflect the long-term durability of crowns, particularly when assessing esthetic alternatives to SSCs placed using the HT. While BFCs demonstrated satisfactory performance in the short term, their risk of perforation may increase beyond one year. Therefore, BFCs may represent a reasonable option for teeth requiring short- to medium-term coverage, such as in uncooperative patients with esthetic concerns or for primary teeth nearing exfoliation, rather than for long-term restorations requiring extended function. Additionally, the relatively narrow age range of the included participants, together with the single-center design and the inclusion of only healthy, cooperative children with mandibular second primary molars, may limit the extrapolation of the findings to younger or older pediatric populations, children with behavioral management difficulties, and primary teeth in different anatomical positions, who may present with different behavioral characteristics, occlusal forces, and oral hygiene practices. Moreover, blinding was not feasible due to the distinct appearance of the two crown types, which could have introduced bias in patient satisfaction assessments and clinical evaluations. In addition, to minimize potential bias, standardized and validated assessment criteria were used, and intra-examiner calibration demonstrated high agreement across all evaluated outcomes. In addition, radiographic assessments were partially validated through independent reassessment, and statistical analysis was performed by a blinded independent statistician. Nevertheless, we recognize that the lack of independent blinded outcome assessment may have influenced subjective measures, and future studies should incorporate independent assessors where feasible. It should be noted that radiographic success was not completely independent of clinical success, as radiographic evaluations were restricted to clinically intact restorations, while teeth with clinical failure were automatically classified as treatment failures. Therefore, the radiographic findings should be interpreted in conjunction with the clinical outcomes. Furthermore, because the sample size calculation was powered specifically for the clinical crown-performance outcome, the study may have had limited power to detect a true difference in radiographic success. Accordingly, the non-significant radiographic findings should be interpreted with caution and should not be considered evidence of equivalence between the two treatment groups. In addition, no formal alpha adjustment was made to account for the two co-primary outcomes, which represents a further limitation when interpreting the findings across both endpoints. Future trials evaluating BFCs should consider powering the study based on the co-primary outcome requiring the largest sample size or applying an appropriate multiplicity adjustment across co-primary endpoints. Because of the modest sample size and the very low frequency of clinical failure events, particularly for crown perforation and crown loss, the reliability and interpretability of a formal longitudinal model for the binary clinical outcomes were considered limited in the present dataset. Therefore, between-group comparisons were performed separately at each prespecified follow-up time point, while within-group changes over time were assessed with each group using Friedman’s test for quantitative/ordinal outcomes and Cochran’s Q test for binary outcomes. The results of the time-specific between-group comparisons should therefore be interpreted as comparisons at the respective follow-up time points rather than as a formal longitudinal treatment-by-time analysis. Future studies with larger sample sizes and a greater number of clinical events should use appropriately powered longitudinal models to account for the correlation between repeated observations and to formally evaluate treatment effects over time.

Another limitation is the retrospective registration of the trial, as participant recruitment commenced before trial registration, although the study protocol, eligibility criteria, and outcome measures were established before data analysis and remained unchanged throughout the study. Despite these limitations, the study also has notable strengths. It is among the first clinical trials to directly compare BFCs and SSCs using the HT, providing evidence that no statistically significant differences in clinical and radiographic success were detected between the two crown types over the one-year follow-up period.

The off-label use of BFCs is justified by the need to address a clinically relevant evidence gap, as SSCs - while effective - have notable esthetic limitations. Given the absence of robust clinical data on BFCs, clinical equilibrium exists, warranting evaluation under controlled conditions with appropriate comparators. The study ensured methodological rigor and ethical transparency through a standardized protocol, meticulous follow-up, and a comprehensive informed consent that was obtained from parents after a thorough explanation of the benefits and risks [14]. 

In conclusion, within the limitations of this study, no statistically significant differences in clinical and radiographic outcomes were detected between BFCs and SSCs placed using the HT during the one-year follow-up period. BFCs were associated with higher child and parental satisfaction and may be considered an acceptable short-term esthetic alternative to SSCs for restoring primary molars. However, perforation occurred exclusively in the BFC group, and its long-term implications for restoration longevity and pulpal health remain uncertain. Therefore, the findings should be interpreted with caution, and further long-term studies are required to evaluate the durability and biological performance of BFCs.

## Data Availability

All data included in this study are available from the corresponding author upon reasonable request.
